# Emerging impact of the long noncoding RNA MIR22HG on proliferation and apoptosis in multiple human cancers

**DOI:** 10.1186/s13046-020-01784-8

**Published:** 2020-12-03

**Authors:** Le Zhang, Cuixia Li, Xiulan Su

**Affiliations:** Clinical Medical Research Center of the Affiliated Hospital, Inner Mongolia Medical University, 1 Tong Dao Street, Huimin District, Inner Mongolia 010050 Hohhot, China

**Keywords:** Long noncoding RNAs, MIR22HG, Tumorigenesis, Therapeutic target

## Abstract

An increasing number of studies have shown that long noncoding RNAs (lncRNAs) play important roles in diverse cellular processes, including proliferation, apoptosis, migration, invasion, chromatin remodeling, metabolism and immune escape. Clinically, the expression of MIR22HG is increased in many human tumors (colorectal cancer, gastric cancer, hepatocellular carcinoma, lung cancer, and thyroid carcinoma), while in others (esophageal adenocarcinoma and glioblastoma), it is significantly decreased. Moreover, MIR22HG has been reported to function as a competitive endogenous RNA (ceRNA), be involved in signaling pathways, interact with proteins and interplay with miRNAs as a host gene to participate in tumorigenesis and tumor progression. In this review, we describe the biological functions of MIR22HG, reveal its underlying mechanisms for cancer regulation, and highlight the potential role of MIR22HG as a novel cancer prognostic biomarker and therapeutic target that can increase the efficacy of immunotherapy and targeted therapy for cancer treatment.

## Background

With the development of genome-wide sequencing technology, there is a deeper understanding of the transcriptomes of organisms. It is currently believed that > 90% of noncoding RNAs (ncRNAs) in the human genome play important biological roles, whereas they were previously considered “transcriptional noise” or “transcriptional waste” [1, 2]. Depending on their length, ncRNAs can be divided into two classes, small noncoding RNAs (ncRNA < 200 nt), including miRNAs, siRNAs, and piRNAs, and long noncoding RNAs (lncRNAs > 200 nt), both of which lack the ability to encode proteins [3]. An increasing number of studies have shown that lncRNAs play important roles in regulating important cell biological functions, such as cell proliferation, apoptosis, migration, invasion, drug resistance and the immune response [4, 5]. In addition, the abnormal expression of lncRNAs, such as MALAT1, HOTAIR, H19 and TUG1, is closely related to the occurrence and development of various malignant tumors [6, 7]. This article summarizes the related research reports on MIR22HG in common tumors, summarizes its biological functions and potential mechanisms in tumors, and provides clues for its application in diagnosis, efficacy and prognosis.

LncRNAs are regulatory RNAs with a length greater than 200 nt and lack protein-coding potential. Increasing evidence has shown that lncRNAs regulate the molecular processes of tumors at the transcriptional, translational, and epigenetic levels. If located in the cytoplasm, lncRNAs may play a regulatory role in “stabilizing RNAs”, “regulating mRNA translation”, acting as “ceRNAs”, “acting as miRNA precursors” or “mediating protein modifications” [8]; if located in the nucleus, lncRNAs play a regulatory role in two ways: “cis-acting” or “trans-acting” [9, 10]. Furthermore, certain lncRNAs show cell- and tissue-specific expression patterns that are critical for their functional analysis and exploration of the potential of lncRNAs as diagnostic, prognostic, and therapeutic targets in cancer [11–18]. Many lncRNAs and their functions or mechanisms need to be further studied, some of which have currently highly attracted the attention of researchers.

In this review, we note that lncRNA NR 028502.1 is located in 17p13.3, a chromosomal region that is frequently deleted or hypermethylated or shows loss of heterozygosity [19, 20]. NR 028502.1 was identified as a lncRNA in the Encyclopedia of DNA Elements (ENCODE) project and is currently annotated as being discovered. It has four different transcripts: MIR22HG-1 (2659 bp, transcript variant 1), MIR22HG-2 (1852 bp, transcript variant 2), MIR22HG-3 (1439 bp, transcript variant 3), and MIR22HG-4 (1356 bp, transcript variant 4). Existing studies have shown that MIR22HG functions as a tumor suppressor in many cancers, such as gastric cancer, colorectal cancer, esophageal cancer, lung cancer and hepatocellular carcinoma. However, in esophageal adenocarcinoma and glioblastoma, MIR22HG acts as a tumor promoter to facilitate tumor progression. Considering the differential expression and significant biological function of MIR22HG, it may have great value for diagnostic, prognostic, and therapeutic cancer research. Therefore, in-depth research on the roles of MIR22HG in different tumors and its possible mechanisms of action will provide new insight into clinical cancer treatment. This article provides an overview of existing research on MIR22HG and highlights its promising clinical application as a potential biomarker for the prevention, diagnosis and treatment of cancer.

## Regulatory mechanisms of MIR22HG in cancer

**Fig. 1:**
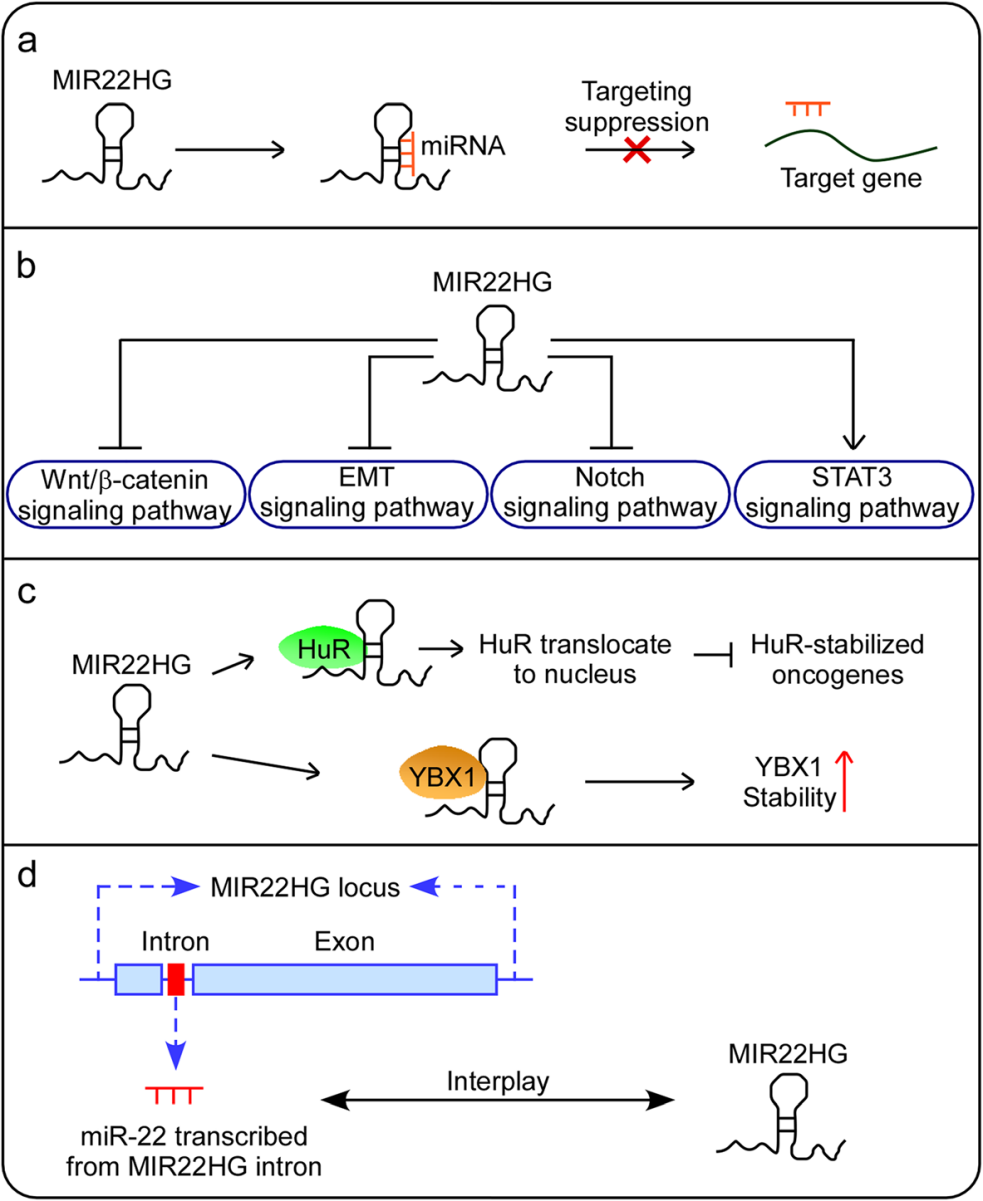
Working mechanisms implicated in the MIR22HG-mediated regulation of cancer progression. **a** MIR22HG acts as a ceRNA. **b** MIR22HG is involved in signaling pathways. **c** MIR22HG interacts with proteins. **d** MIR22HG interplays with miRNAs as a host gene

Studies so far suggest that lncRNAs play critical roles in both normal cellular functions and diseases, including cancer. MIR22HG, a well-studied lncRNA, has been shown to function as a master regulator in diverse malignancies and thus can play a critical role in various aspects of carcinogenesis, including proliferation, apoptosis, invasion, and metastasis (Table 1). Importantly, the aberrant expression of MIR22HG is significantly associated with important clinical characteristics, such as advanced tumor size, stage, TNM stage and overall survival in various kinds of human cancer (Table 2). Various mechanisms have been implicated in the MIR22HG-mediated regulation of cancer progression; for example, MIR22HG has been reported to function as a competitive endogenous RNA (ceRNA) (Fig. 1a), be involved in signaling pathways (Fig. 1b), interact with proteins (Fig. 1c) and interplay with miRNAs as a host gene (Fig. 1d).

**Table 1:** Functional characterization of MIR22HG in various tumors

Tumor type	Expression	Role	Functional role	Related genes and pathways	References
thyroid carcinoma	down regulated	tumor suppressor	cell proliferation, migration, invasion and apoptosis	Hippo signaling pathway, miR-24-3p and p27kip1	[21, 22]
hepatocellular carcinoma	down regulated	tumor suppressor	cell proliferation, migration, and invasion	miR-22-3p, HMGB1, HuR, miR-10a-5p, NCOR2, β-catenin and EMT	[23, 24]
endometrial carcinoma	down regulated	tumor suppressor	cell proliferation, apoptosis and the cell cycle	miR-141-3p and DAPK1	[25]
cholangiocarcinoma	down regulated	tumor suppressor	cell proliferation, migration, and invasion	Wnt/β-catenin signaling pathway, β‐catenin, cyclin D1 and c‐myc	[26]
colorectal cancer	down regulated	tumor suppressor	cell proliferation and migration	SMAD2, SMAD4, TGFβ signaling pathway, EMT, and CD8A	[27]
gastric cancer	down regulated	tumor suppressor	cell proliferation, migration, and invasion	Notch2 signaling pathway and HEY1	[28]
esophageal adenocarcinoma	up regulated	tumor promoter	cell proliferation, migration, invasion and apoptosis	STAT3, c-Myc and p-FAK	[29]
non-small cell lung cancer	down regulated	tumor suppressor	cell proliferation, migration, and invasion	Ybx1, MET, and p21	[30]
glioblastoma	up regulated	tumor promoter	cell proliferation and invasion	MiR-22-3p/miR-22-5p, SFRP2/PCDH15, ACIL6JTK and Wnt/β-catenin signaling pathway	[31]

**Table 2:** Clinical significance of MIR22HG in various human tumors

Cancer type	Clinicopathological features	References
thyroid carcinoma	Low MIR22HG expression was related to tumor size (*P* = 0.015), TNM stage (*P* = 0.022) and poor overall survival (*P* = 0.030).	[21]
	Low MIR22HG expression was significantly related to the lymph node metastasis status (*P* < 0.01), the residual tumor status (*P* < 0.05), N stage (*P* < 0.05), tumor grade (*P* < 0.001) and T stage (*P* < 0.001) while high MIR22HG expression was significantly correlated with the disease recurrence rate (*P* < 0.01), overall survival time (*P* = 0.0665) and disease-free survival time (*P* < 0.05) in TC by analyzing TCGA, the GSE29265, GSE33630, and GSE55091 public datasets.	[22]
hepatocellular carcinoma	Low MIR22HG expression was associated with short overall survival (*P* = 0.045) and poor disease-free survival (*P* = 0.036).	[23]
	Patients with high MIR22HG expression exhibited better overall survival (145-patient cohort: *P* = 0.001; TCGA cohort: *P* = 0.015) and disease-free survival (145-patient cohort: *P* = 0.042; TCGA cohort: *P* = 0.003) than those with low MIR22HG expression.	[24]
cholangiocarcinoma	Low MIR22HG expression was positively correlated with advanced clinical stage (TNM) (*P* = 0.039), large tumor size (*P* = 0.002), lymph node metastasis (*P* = 0.0001), and poor overall survival (*P* = 0.020).	[26]
colorectal cancer	Low MIR22HG expression was significantly associated with poor overall survival (*P* = 0.0008) and disease-free survival (*P* = 0.0009).	[27]
gastric cancer	Low MIR22HG expression indicated a low 5-year overall survival rate (*P* < 0.05).	[28]
non-small cell lung cancer	Low MIR22HG expression was correlated with poor patient survival (*P* = 0.003) in an independent UM cohort including 101 LUAD tissues and 27 normal lung tissues.	[30]
glioblastoma	High MIR22HG expression was associated with patient age (*P* < 0.001), Karnofsky Performance Status score (*P* < 0.001), advanced tumor grade and poor overall survival (*P* < 0.05).	[31]

### Function as a ceRNA

One of the most well-characterized mechanisms of lncRNAs is functioning as a ceRNA or “sponge” for miRNAs. ceRNAs are involved in posttranscriptional regulation, as they compete with miRNAs through the same miRNA sequence during RNA transcription to regulate the expression of downstream target genes [32–34]. Mounting evidence has demonstrated that ceRNAs play a vital role in cancer progression [35–37].

**Fig. 2:**
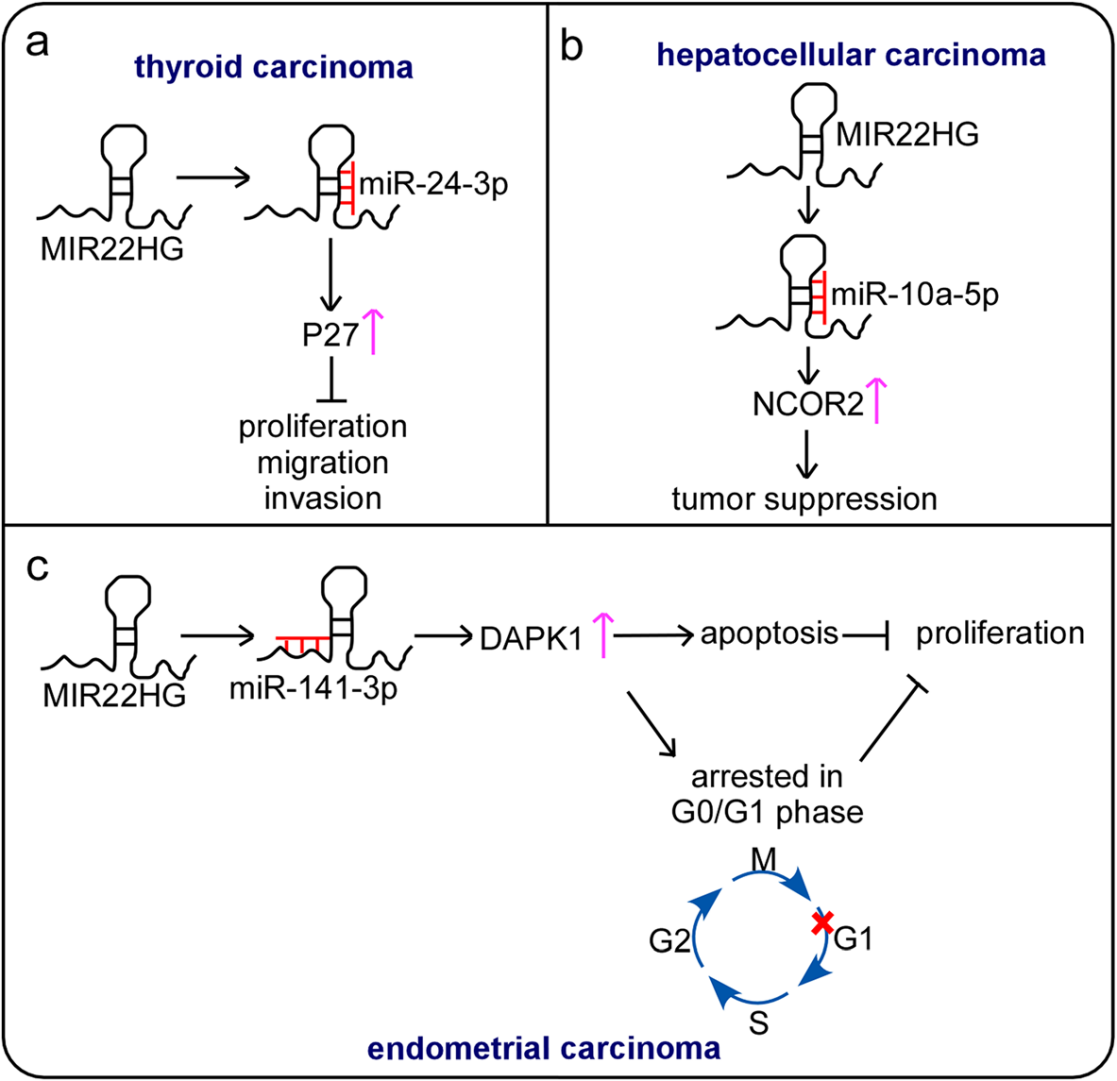
MIR22HG functions as a ceRNA in human cancers.** a** MIR22HG functions as an endogenous sponge of miR-24-3p to increase the expression of p27, suppressing the proliferation, migration and invasion of TC. **b** MIR22HG acts as a ceRNA to bind with miR-10a-5p to increase the expression of NCOR2 and inhibit HCC progression. **c** MIR22HG functions as a ceRNA to bind with miR-141-3p to upregulate DAPK1 expression levels, resulting in EC cell proliferation inhibition

More recently, MIR22HG has also emerged as exhibiting ceRNA functions in many cancer types, such as thyroid carcinoma (TC), endometrial carcinoma (EC) and hepatocellular carcinoma (HCC) (Fig. 2). Chen et al. revealed that MIR22HG was downregulated in TC tumor tissues compared with nontumor tissues using 40 pairs of papillary thyroid carcinoma tissues through qRT-PCR detection. Further biological function studies have demonstrated that MIR22HG suppresses the growth, migration and invasion of TC cells. Systematically, MIR22HG acts as a ceRNA to upregulate p27kip1 by directly binding with miR-24-3p, which inhibits the malignant phenotype of TC cells [21] (Fig. 2a). Moreover, the authors reported that low MIR22HG expression was related to tumor size (*P* = 0.015), TNM stage (*P* = 0.022) and poor overall survival (*P* = 0.030) by qRT-PCR detection. Another study by Qin et al. verified these findings. They reported that the downregulation of MIR22HG was significantly related to the lymph node metastasis status (*P* < 0.01), the residual tumor status (*P* < 0.05), N stage (*P* < 0.05), tumor grade (*P* < 0.001) and T stage (*P* < 0.001) while high MIR22HG expression was significantly correlated with the disease recurrence rate (*P* < 0.01), overall survival time (*P* = 0.0665) and disease-free survival time (*P* < 0.05) in TC by analyzing TCGA, GSE29265, GSE33630, and GSE55091 public database. Coexpression, Gene Ontology (GO) and Kyoto Encyclopedia of Genes and Genomes (KEGG) pathway analyses revealed that MIR22HG was involved in regulating apoptosis, transcription, the cell cycle, and Hippo signaling [22].

Wu et al. found that MIR22HG was significantly decreased in 120 HCC tissues compared with adjacent nontumor liver tissues by employing qRT-PCR. Furthermore, low MIR22HG expression was associated with short overall survival (*P* = 0.045) and poor disease-free survival (*P* = 0.036). These results implicated the potential role of MIR22HG as a diagnostic and prognostic biomarker to improve HCC patient outcomes. Functional experiments demonstrated that the knockdown of MIR22HG promoted the growth, migration and invasion of HCC cells. It has also been reported that miR-10a functions as an oncogene or tumor suppressor depending on the context [38–41]. Mechanistically, miR-10a-5p can be a downstream target of MIR22HG. By acting in this manner as a ceRNA, MIR22HG directly binds to miR-10a-5p and increases NCOR2 expression to inhibit the proliferation, migration and invasion of HCC cells [23] (Fig. 2b).

Not coincidentally, Cui’s team confirmed the critical role by which MIR22HG acts as a ceRNA to modulate the proliferation, apoptosis and cell cycle of EC cells [25] (Fig. 2c). MIR22HG overexpression significantly reduced miR-141-3p expression in EC cells. Subsequently, MIR22HG increased DAPK1 expression by targeting miR-141-3p, thus inhibiting cell proliferation via G1 arrest of the cell cycle and promoting the apoptosis of EC cells. In summary, the MIR22HG-regulated miR-141-3p/DAPK1 axis may be a new therapeutic target for the treatment and prevention of EC.

### Involvement in signaling pathways

**Fig. 3:**
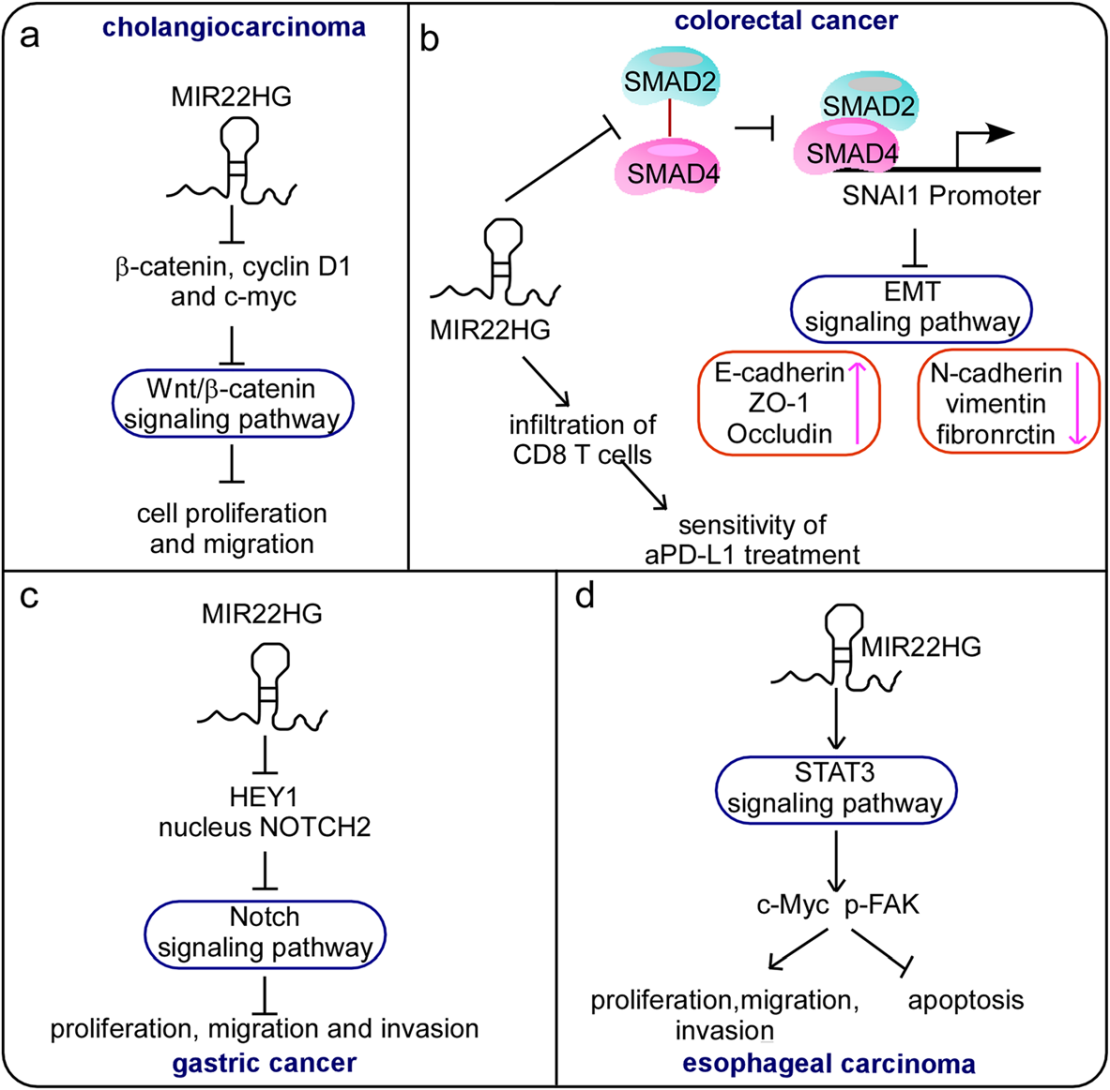
MIR22HG is involved in signaling pathways that affect cancer progression. **a** MIR22HG negatively regulates the Wnt/β-catenin signaling pathway by downregulating the expression of β-catenin, cyclin D1 and c-myc to inhibit cell proliferation and migration in CCA. **b** MIR22HG blocks the SMAD complex, preventing its binding to the promoter of SNAI1 and further suppressing the EMT signaling pathway. **c** MIR22HG inhibits the Notch signaling pathway by downregulating nuclear Notch2 and HEY1 expression in GC. **d** MIR22HG activates the STAT3 signaling pathway to promote the proliferation, migration and invasion of ESCA

Signaling pathways coordinate communication to enable cells to respond to intracellular or extracellular stimuli. There are many different signaling pathways that contribute to development and cellular homeostasis [42–44]. In diseases, especially cancer, aberrant signaling has been identified as a key mechanism of cancer progression and metastasis [45]. Growing evidence suggests that the MIR22HG-mediated dysregulation of signaling pathways is central to many different types of cancer (Fig. 3).

#### Wnt/β-catenin signaling pathway

Wnt/β-catenin signaling is an evolutionarily conserved regulatory pathway that has diverse roles in governing cell fate, proliferation, migration, polarity, and death [46, 47]. Accumulating evidence has shown that inappropriate activation of the Wnt/β-catenin pathway is an important mechanism for cancer progression [48–51], and therapeutics targeting Wnt/β-catenin signaling have shown promising clinical applications [52–54].

A report by Hu et al. showed that MIR22HG was downregulated in cholangiocarcinoma (CCA) issues and cell lines by RT-qPCR. The low expression of MIR22HG in CCA tissues was positively correlated with an advanced clinical stage (TNM) (*P* = 0.039), large tumor size (*P* = 0.002), lymph node metastasis (P = 0.0001), and poor overall survival (P = 0.020) [26]. The Wnt/β-catenin signaling pathway is involved in the regulation of downstream c-myc, cyclin D1 and other oncogenes that play an important role in tumor cell proliferation and apoptosis [55, 56]. Importantly, the proto-oncogene c-myc is a vital cell cycle regulator in DNA synthesis and cell cycle progression [57, 58]. Hu et al. reported that MIR22HG negatively regulated the Wnt/β-catenin signaling pathway by downregulating the expression of β-catenin, cyclin D1 and c-myc, leading to the inhibition of cell proliferation, migration and invasion in CCA cells. Further in vivo studies in which mouse subcutaneous xenografts were used confirmed that MIR22HG suppresses CCA tumorigenesis (Fig. 3a). In conclusion, MIR22HG may be a novel target for diagnosis and therapy in CCA [26].

#### Epithelial-mesenchymal transition (EMT) signaling pathway

EMT is a biological process in which epithelial cells lose their characteristic apical-basal polarity and markers while acquiring the characteristics of mesenchymal cells, with high migration and invasion abilities [59]. In recent years, EMT has become a hot spot of cancer research because of its roles in the initial process of tissue carcinogenesis. EMT markers are closely associated with the EMT process and EMT-related migration, invasion, proliferation, antiapoptosis, stemness, and tumor radio/chemosensitivity of cancer cells [60, 61].

Xu et al. reported that MIR22HG was downregulated in colorectal cancer (CRC) tissues and cells compared with normal tissues and cells, as determined by qRT-PCR [27]. Functional analyses revealed that MIR22HG inhibits CRC cell proliferation, migration and invasion in vitro. Then, the authors employed mouse subcutaneous xenograft models and three metastasis models, namely, an intestine metastasis mouse model, a lung metastasis mouse model and an orthotopic hepatic metastasis mouse model, to examine the effect of MIR22HG on tumor growth and metastasis in vivo. The results indicated that tumors formed by MIR22HG-overexpressing cells were smaller and weighed less than tumors formed by control cells and exhibited fewer metastatic nodules and sparse and small metastatic foci. Moreover, they discovered that MIR22HG overexpression inhibited the EMT process, and MIR22HG silencing produced the opposite effect. Silencing MIR22HG decreased the expression of epithelial markers (E-cadherin, ZO-1 and Occludin) and increased the expression of mesenchymal markers (N-cadherin, vimentin and fibronectin). Systematically, MIR22HG inhibits the interaction between SMAD2 and SMAD4 of the TGFβ pathway. Blocking the formation of the SMAD complex also prevents its binding to the promoter of SNAI1 and further suppresses the EMT process (Fig. 3b). These results imply that the MIR22HG-mediated SMAD2/4-SNAI1 axis plays a critical role in CRC progression by regulating EMT signaling pathways. Importantly, an increasing body of evidence has shown that the TGFβ pathway can reshape the immune environment of tumors [62, 63]. The authors further investigated the function of MIR22HG in immune using syngeneic immunocompetent mouse model C57BL/6 [27]. The combination of MIR22HG and aPD-L1 enhanced sensitivity to immunotherapy, suppressed tumor growth, and prolonged the overall survival of mice by promoting CD8 T cell infiltration and facilitating immunotherapy in CRC. These observations indicate the potential application of MIR22HG in CRC immunotherapy by acting as a tumor suppressor.

#### Notch signaling pathway

The Notch family is a highly conserved and important transmembrane signaling protein family involved in cell development, differentiation, proliferation and apoptosis [64]. The role of Notch signaling in cancer is highly context-dependent [65]. It can act as an oncogene in T cell acute lymphoblastic leukemia (T-ALL), breast cancer, and ovarian cancer, while it can also exerts an important tumour-suppressor function in other cancers, such as HCC, forebrain glioma, and head and neck squamous cell carcinoma (HNSCC) [66, 67]. Given its complicated role in tumorigenesis, the Notch signaling pathway will need to be explored in more detail.

Li et al. reported that MIR22HG was downregulated in 43 pairs of human gastric cancer (GC) tissues compared to 21 pairs of matched normal tissues by RT-qPCR [28]. Clinicopathological analysis showed that low MIR22HG expression correlated with poor 5-year overall survival (P < 0.05) in GC patients. The authors found that upregulated MIR22HG may suppress GC cell proliferation, invasion and migration in vitro. In addition, a mechanistic investigation revealed that MIR22HG negatively regulates NOTCH2 signaling by downregulating the expression of HEY1 and nuclear NOTCH2 [28]. MIR22HG knockdown did not influence the expression of NOTCH2 but markedly enhanced that of nuclear NOTCH2. These data suggest that MIR22HG inhibits GC progression by attenuating NOTCH2 signaling (Fig. 3c).

#### STAT3 signaling pathway

The STAT3 signaling pathway has been demonstrated to be important for cancer progression. First, it transduces signals from numerous receptor and nonreceptor tyrosine kinases that are frequently activated in cancer cells [68]. Second, as a transcription factor, STAT3 regulates the expression of many oncogenes, such as c cyclin B1, CDC2, p53, MCL-1, survivin, VEGF, BCL2 and BAX [69]. Third, growing evidence suggests that STAT3 signaling plays a crucial role in the suppression of tumor immune surveillance and may be a candidate therapeutic target for multiple antitumor immune responses [70]. In addition, cumulative research has shown that the STAT3 signaling pathway is activated in a variety of tumors, such as breast cancer [71], melanoma [72], brain tumors [73], and GC [74, 75], and promotes cell growth and survival, angiogenesis, migration, invasion or metastasis [76].

Su et al. proved that MIR22HG was markedly overexpressed in esophageal cancer (ESCA) tissues through analyzing the TCGA database [29]. Cell experiments revealed that MIR22HG knockdown inhibited cell proliferation, migration and invasion in 3 esophageal adenocarcinoma (EAC) cell lines (OE33, OE19 and FLO-1). Moreover, MIR22HG knockdown suppressed the activity of the STAT3 signaling pathway by downregulating STAT3, c-Myc and p-FAK protein expression, thus inducing apoptosis in EAC cells. These findings revealed a novel MIR22HG-mediated regulatory mechanism of the STAT3 signaling pathway in EAC and may provide new insights into developing lncRNA-based therapies for this cancer (Fig. 3d).

### Interactions with proteins

Another common mechanism by which lncRNAs mediate their functions is through interactions with proteins. LncRNAs can function as protein decoys to recruit or sequester proteins or act as scaffolds linking different proteins, either coordinately or in a complex [77]. LncRNA-protein interactions exert essential functions in posttranscriptional gene regulation, such as splicing, translation, and signaling, thus participating in the progression of various diseases, including cancer [78, 79].

**Fig. 4:**
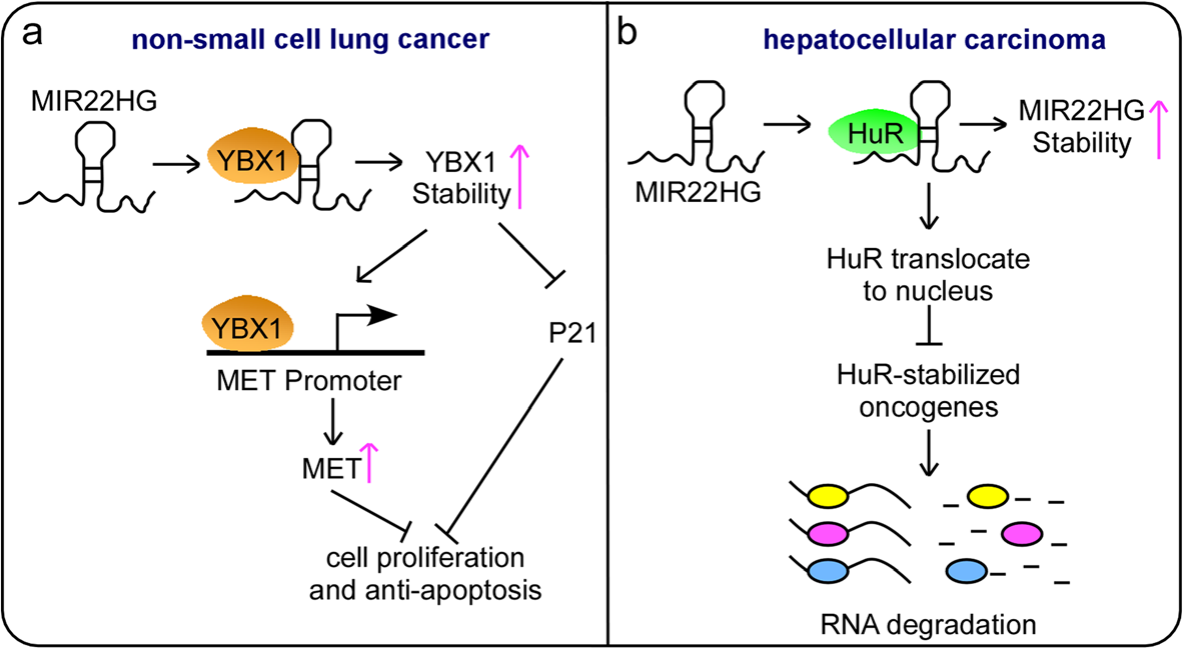
MIR22HG inhibits tumor progression through interactions with proteins.** a** MIR22HG interacts with the YBX1 protein, increasing its stability, leading to the upregulation of MET expression and the inhibition of p21 expression, thus suppressing the proliferation and antiapoptosis of NSCLC cells. **b** MIR22HG specifically interacts with HuR to increase MIR22HG stability and regulate its subcellular localization, resulting in the degradation of HuR-stabilized oncogenes such as β-catenin, CCNB1, HIF1A, BCL2, COX2, and C-FOS

Su et al. analyzed Seo, TCGA and UM RNA-Seq datasets and found that MIR22HG was significantly downregulated in lung adenocarcinoma (LUAD) tissues compared to normal lung tissues. In addition, a high expression level of MIR22HG was significantly correlated with favorable patient outcomes in two independently published LUAD microarray datasets: Okayama et al. (P = 0.02) and Shedden et al. (P = 0.02). The authors employed RT-qPCR to confirm that MIR22HG expression levels were significantly lower in LUAD tissues than in normal lung tissues (P < 0.001) and that low MIR22HG expression was correlated with poor patient survival (P = 0.003) in an independent UM cohort including 101 LUAD tissues and 27 normal lung tissues. MIR22HG overexpression suppresses cell proliferation and invasion and induces cell cycle arrest in non-small cell lung cancer (NSCLC) cells. Mechanistically, MIR22HG interacts with and stabilizes YBX1. The MIR22HG-YBX1 complex can bind to the promoter of MET and promote MET expression at both the mRNA and protein levels and inhibit the expression of p21, a potential target of YBX1, leading to the inhibition of proliferation and promotion of apoptosis (Fig. 4a). The dual role of p21 in the progression of cancer may depend on the cell type, p21 location and p53 status [80–82]. Therefore, in different tumors, p21 may have divergent functions. Upregulated p21 is associated with poor survival in patients with glioma and prostate, cervical, ovarian, and esophageal cancers. However, the opposite is observed in other tumors, such as breast, gastric, and ovarian cancers [30]. In this study, the authors revealed the biological role of p21 as a potent oncogene that promotes tumor growth in NSCLC and shed light on a new therapeutic strategy for the regulatory mechanism of p21 mediated by lncRNA-protein interactions [30].

Zhang’s team measured MIR22HG levels in a 52-patient cohort by qRT-PCR (*P* < 0.001), analyzed TCGA and the GSE14520 cohorts (*P* < 0.001 for both cohorts) and revealed that MIR22HG was comparatively expressed at low levels in HCC tissues. Kaplan-Meier analysis of overall survival and disease-free survival (log-rank) in the 145-patient cohort and the TCGA cohort revealed that patients with high MIR22HG expression exhibited better overall survival (145-patient cohort: *P* = 0.001; TCGA cohort: *P* = 0.015) and disease-free survival (145-patient cohort: *P* = 0.042; TCGA cohort: *P* = 0.003) than those with low MIR22HG expression. Functionally, biological studies have revealed that the overexpression of MIR22HG dramatically inhibits cell proliferation, migration and invasion in vitro. Moreover, the overexpression of MIR22HG significantly inhibits tumor growth and metastasis in vivo according to mouse subcutaneous xenograft models and a lung metastasis mouse model. Conversely, the silencing of MIR22HG promotes cell proliferation both in vitro and in vivo. Mechanistically, MIR22HG can interact with the human antigen R (HuR) protein, an RNA-binding protein positively associated with malignant aggressiveness [83–85]. This lncRNA-protein interaction increases MIR22HG stability and regulates the subcellular localization of HuR, resulting in the decreased expression of HuR-stabilized oncogenes such as β-catenin, CCNB1 (encoding cyclin B1), HIF1A (encoding hypoxia-inducible factor-1α), BCL2 (encoding apoptosis regulator Bcl2), COX2 (encoding cyclooxygenase COX2), and C-FOS (encoding the nuclear phospho-protein c-Fos) and thereby inhibiting the proliferation, invasion and migration of HCC (Fig. 4b). Importantly, their investigation may help identify potential biomarkers that can improve the diagnosis and treatment of HCC [24].

### Interplay with miRNAs as a host gene

**Fig. 5:**
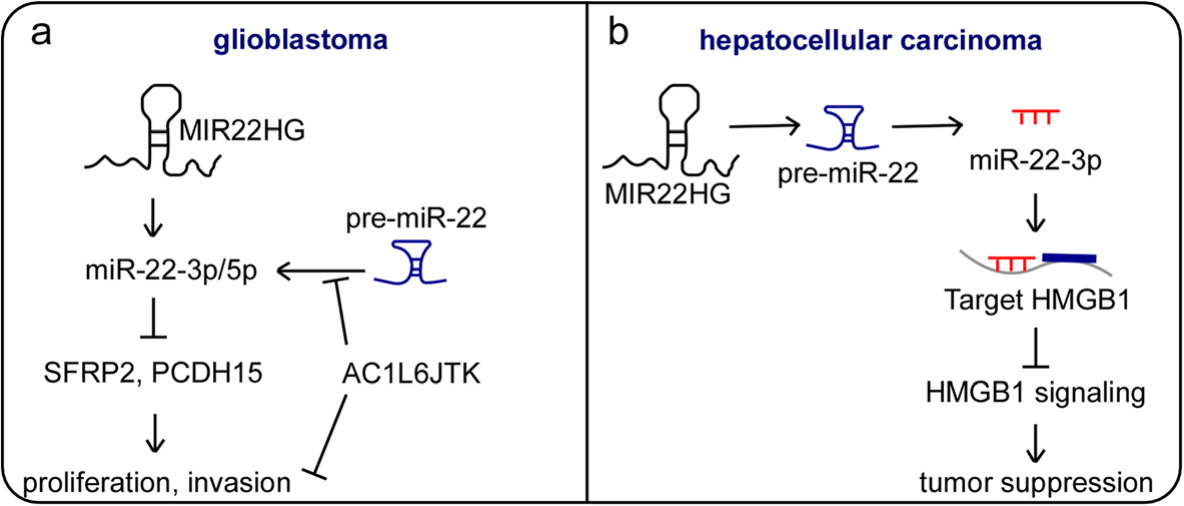
MIR22HG interacts with miR-22 as a host gene.** a** MIR22HG, as a host gene of miR-22, upregulates the expression of miR-22-3p/5p, leading to the attenuated expression of its two direct targets, SFRP2 and PCDH15, thus promoting GBM cell proliferation and invasion. AC1L6JTK, a specific small-molecule inhibitor, efficiently suppresses tumor growth in vivo by blocking the processing of pre-miR-22 into mature miR-22. **b** MIR22HG markedly upregulates miR-22-3p expression levels and drives miR-22-3p to target HMGB1, leading to the deactivation of HMGB1 signaling

The regulatory relationship between miRNAs and their host genes provides another mechanism for lncRNA-mediated gene expression. A previous study described MIR22HG as a host gene of miR-22 [86]. Two independent studies examined whether MIR22HG functions as the host gene of miR-22 in cancer progression (Fig. 5). Han et al. revealed that the expression of MIR22HG was higher in glioblastoma (GBM) and glioma stem-like cells than in normal neural stem cells and that increased MIR22HG was correlated with poor overall survival (*P* < 0.0001) in an analysis of a TCGA dataset. Silencing MIR22HG inhibited GBM cell proliferation and invasion in vitro. In vivo studies in which mouse subcutaneous xenograft and brain orthotopic xenograft models were used have revealed that MIR22HG inhibits tumor growth and metastasis. A mechanistic analysis revealed that MIR22HG, as a host gene of miR-22, is strongly associated with the expression of miR-22-3p and miR-22-5p. Rescue experiments showed that the overexpression of miR-22 is sufficient to restore the MIR22HG depletion-induced inhibition of GBM cell proliferation and invasion. Silencing MIR22HG resulted in the loss of miR-22-3p and miR-22-5p, which upregulated the expression of their direct targets SFRP2 and PCDH15, leading to the inhibition of GBM progression. AC1L6JTK, a specific small-molecule inhibitor, efficiently suppresses tumor growth in vivo by blocking the processing of pre-miR-22 into mature miR-22. These data indicate that the interplay between miR-22 and its host gene MIR22HG might be a potential target for patients with GBM through pharmacological blockade [31] (Fig. 5a). Another independent study confirmed the important role of this interaction in cancer. In addition to its interaction with HuR, Zhang et al. also found that increased MIR22HG could markedly upregulate miR-22-3p expression levels. Additionally, MIR22HG drives miR-22-3p to target HMGB1, leading to the deactivation of HMGB1 signaling. These data demonstrate that miR-22-3p and its host gene MIR22HG are coexpressed and functionally coordinated in HCC [24] (Fig. 5b).

## Therapeutic implications of MIR22HG in cancer

Currently, MIR22HG is included in a long list of lncRNAs that are mechanistically linked to the progression and prognosis of several types of cancer. It has been reported that MIR22HG is downregulated in GC, HCC, NSCLC, TC, CCA, and CRC and that the low expression of MIR22HG is significantly associated with poor overall survival (Table 2). Given the differential expression of MIR22HG in cancer, MIR22HG might be a novel biomarker for cancer diagnosis and prognosis. Not coincidentally, in terms of lncRNAs functioning as biomarkers, one of the most well-known examples is PCA3, a prostate-specific lncRNA previously named DD3 [87]. Considering that PCA3 is prostate tissue-specific and highly overexpressed in prostate cancer (PC) tissues compared with benign tissues, it has attracted the interest of academic researchers who validated the potential role of PCA3 as a biomarker for PC diagnosis [88–92]. Finally, in 2012, the US Food and Drug Administration (FDA) approved Progensa PCA3 as an aid for repeat biopsy decisions in men with a previous negative biopsy [93]. PCA3 is a successful example translated from an academic research laboratory into clinical practice, providing a promising future for lncRNA-based clinical applications.

Overwhelming evidence supports the proproliferative and antiapoptotic roles of MIR22HG in cancer. In addition, MIR22HG exhibits extensive mechanistic diversity to carry out its functional roles; therefore, it may represent a novel target to overcome cancer. Currently, there are several clinical trials involving lncRNAs as novel biomarkers or cancer therapies (database: http://clinicaltrials.gov). Two clinical trials sponsored by Assiut University will evaluate the clinical utility of detecting the expression of the lncRNA CCAT1 in the diagnosis of CRC patients and its relation to tumor stage (NCT04269746) and investigate the lncRNAs HOTAIR and Midkine as biomarkers in TC. Furthermore, two other clinical trials are recruiting patients to validate lncRNAs as biomarkers for the detection and prognosis of lung cancer (NCT03830619) and high-grade serous ovarian cancer (HGSOC) (NCT03738319). A trial sponsored by Fudan University is currently enrolling subjects to compare the efficacy and safety between docetaxel combined with doxorubicin (epirubicin) and cyclophosphamide followed by gemcitabine combined with cisplatin and doxorubicin (epirubicin) combined with cyclophosphamide followed by docetaxel for high-risk triple-negative breast cancer predicted by the mRNA-lncRNA integrated signature and to validate the efficacy of the signature (NCT02641847). Importantly, the development of new RNA biology technologies and approaches offers more opportunities for lncRNA-targeted clinical applications. For instance, small interfering RNAs (siRNAs) and antisense oligonucleotides (ASOs) are the most common RNA-targeted therapies. To efficiently and safely target RNA, some chemical modifications, such as nucleoside moieties, morpholinos, and peptide nucleic acids, can be introduced [94]. The biological function of MIR22HG in cancer will need to be explored in more detail, and its possible relevance to cancer therapeutic targets will also need to be examined.

Another potentially exciting use for MIR22HG may be in the area of immunotherapy. Immunotherapy is a type of cancer treatment that helps the immune system fight cancer. Checkpoint inhibitors are a type of immunotherapy that takes the brakes off the immune system and helps it recognize and attack cancer cells [95]. Although checkpoint inhibitors have made large breakthroughs in cancer treatment, remarkable responses are currently limited to a minority of patients and indications. Thus, one major concern is how we can enhance the efficiency and response rate of checkpoint inhibitors. Notably, checkpoint inhibitors do not work directly on the tumor, but their efficiency depends on whether the patient’s own T cells can infiltrate the tumor [96]. Xu and colleagues revealed that MIR22HG expression is significantly correlated with CD8A and that the overexpression of MIR22HG triggers T cell infiltration, which plays a central role in coordinating distinct types of immune responses. In this regard, MIR22HG may be a novel biomarker to predict the immunotherapy response. Further in vivo studies confirmed that mice treated with MIR22HG and PD-L1 blockade had smaller size and lower weight tumor and responded more positively to anti-PD-L1 immunotherapy than untreated mice. These findings in mice also correlate with studies of T cell infiltration as a key limiting factor for efficacious cancer immunotherapy.

## Conclusion

Up-to-date studies have provided a comprehensive overview showing that MIR22HG is recognized as a regulator of cancer-influencing proliferation, apoptosis, and migration. MIR22HG drives the cancer phenotype through the dysregulation of oncogenic and tumor suppressive gene networks via the variety of mechanisms discussed above. Of note, its aberrant expression is closely correlated with clinicopathological parameters, such as lymphatic metastasis, tumor stage, tumor size and overall survival, providing a great opportunity as a cancer prognostic biomarker. Furthermore, with diverse modulatory mechanisms, MIR22HG has advantages that support its potential as a therapeutic target. To date, research on the mechanism of MIR22HG has made some progress but remains mainly in the preclinical stage. Future investigations will be necessary to explore the precise molecular regulatory mechanisms of MIR22HG in carcinogenesis and cancer progression to translate MIR22HG from basic research into the clinic as early as possible.

## Data Availability

Not applicable.

## Abbreviations

ncRNAs: Noncoding RNAs
lncRNA: Long noncoding RNA
ceRNA: Competing endogenous RNA
TC: Thyroid carcinoma
EC: Endometrial carcinoma
HCC: Hepatocellular carcinoma
CCA: Cholangiocarcinoma
EMT: Epithelial-mesenchymal transition
CRC: Colorectal cancer
T-ALL: T cell acute lymphoblastic leukemia
HNSCC: Head and neck squamous cell carcinoma
GC: Gastric cancer
ESCA: Esophageal carcinoma
EAC: Esophageal adenocarcinoma
LUAD: Lung adenocarcinoma
NSCLC: Non-small cell lung cancer
HuR: Human antigen R
CCNB1: Cyclin B1
HIF1A: Hypoxia-inducible factor-1α
COX2: Cyclooxygenase COX2
GBM: Glioblastoma
PC: Prostate cancer
FDA: Food and Drug Administration
PTC: Papillary thyroid carcinoma
siRNAs: Small interfering RNAs
ASOs: Antisense oligonucleotides
